# Antidepressants and Lung Toxicity: A Narrative Review

**DOI:** 10.7759/cureus.89263

**Published:** 2025-08-02

**Authors:** Ariana Azimi Shooshtari, Elakiya Anjali Jayaraman, Ramesh Kesavan, Siva Sarva, Skantha Manjunath, Azad Patel

**Affiliations:** 1 College of Liberal Arts, University of Texas at Austin, Austin, USA; 2 Pulmonary and Critical Care Medicine and Internal Medicine, HCA Houston Healthcare Kingwood/University of Houston, Kingwood, USA; 3 Pulmonary and Critical Care Medicine, HCA Houston Healthcare Kingwood/University of Houston, Kingwood, USA

**Keywords:** acute respiratory distress syndrome (ards), antidepressant, drug-induced pneumonia, lung injury, psychotropic drugs

## Abstract

Pulmonary toxicity is a serious yet frequently under-recognized complication of antidepressant therapy. With the continued rise in prescriptions, awareness of potential respiratory adverse effects is crucial. This review outlines documented cases of lung injury linked to various antidepressant classes, including selective serotonin reuptake inhibitors (SSRIs), serotonin-norepinephrine reuptake inhibitors (SNRIs), atypical antidepressants, serotonin modulators, tricyclic antidepressants (TCAs), and monoamine oxidase inhibitors (MAOIs). A wide range of manifestations, such as interstitial lung disease, eosinophilic pneumonia, acute respiratory distress syndrome, and alveolar hemorrhage, occurring at both standard dosages and in overdose situations, have been documented.

Clinicians should remain vigilant for respiratory symptoms emerging during treatment and pursue timely evaluation and management to minimize the risk of long-term pulmonary complications and improve clinical outcomes.

## Introduction and background

Psychological disorders are a rapidly growing global crisis. The current point prevalence rate of major depressive disorder (MDD) indicates an affliction of approximately 3.8% of the global population and an estimated 5% of all adults. Within the United States, around 8.3% of adults are diagnosed with MDD, significantly exceeding the global average. In response, selective serotonin reuptake inhibitors (SSRIs) are most commonly prescribed in global treatment. Other classes of antidepressants may be prescribed, including serotonin-norepinephrine reuptake inhibitors (SNRIs) and atypical antidepressants [1,2]. 

While generally effective towards MDD, commonly prescribed antidepressants may yield various side effects, including more common symptoms of nausea, weight gain, or headaches. However, serious complications of pulmonary toxicity have also been noted. This literature review investigates and summarizes reported studies of lung toxicity associated with psychotropic medications. 

## Review

Methodology

We conducted a comprehensive literature search using PubMed to identify articles related to pulmonary toxicity associated with individual antidepressant medications. The search was performed using combinations of the following keywords: "pulmonary toxicity", "lung injury", "interstitial lung disease", "pneumonitis", and the generic name of each antidepressant (e.g., "sertraline", "amitriptyline", "venlafaxine", etc.). Filters were applied to include only articles published in English and involving human subjects. The search covered literature published from January 1980 to June 2025.

In parallel, we reviewed the Pneumotox database (www.pneumotox.com), a specialized resource cataloging drug-induced respiratory disorders, to identify additional reports of pulmonary toxicity linked to antidepressants. Data from both sources were cross-referenced to ensure completeness.

Inclusion criteria were case reports, case series, clinical trials, and observational studies that documented pulmonary adverse effects attributed to antidepressants. Articles were excluded if the association with pulmonary toxicity was unclear or if the diagnosis was confounded by comorbid conditions or co-medications. As the included studies were primarily descriptive, established risk of bias tools were not applicable; however, each study was qualitatively assessed for diagnostic certainty.

Selective serotonin reuptake inhibitors (SSRIs)

SSRIs are the most widely prescribed class of antidepressants in the United States [3]. Due to their widespread use, SSRIs have undergone extensive research examining their potential adverse effects on various organ systems, including cardiovascular, pulmonary, and reproductive systems [4]. Therapeutic effects may take up to six weeks to manifest and are often followed by an increase in dosage by a clinician to achieve clinical benefit.

*Citalopram* 

Citalopram is an SSRI with a half-life of approximately 35 hours, exhibiting an estimated 80% oral bioavailability and reaching peak plasma concentrations typically within four hours [5]. 

Citalopram has been associated with severe pulmonary complications in overdose scenarios. Case reports have documented the development of hypoxemia and acute respiratory distress syndrome (ARDS) following acute citalopram toxicity [6,7].

Acute lung injury may be attributed to serotonin's direct impact on pulmonary endothelial and epithelial cells, resulting in increased permeability of the alveolar-capillary barrier. Additionally, serotonin's central effects may lead to respiratory depression through the suppression of the respiratory drive [6]. 

Fluoxetine

Fluoxetine has a half-life of 2-4 days, while its active metabolite, norfluoxetine, has a significantly longer half-life of 7-9 days [8]. As a result, fluoxetine remains in the body for several weeks after discontinuation, potentially resulting in delayed adverse effects. Patients may present with progressive dyspnea and/or cough [9-11].

Several case reports have documented fluoxetine-induced interstitial lung disease (ILD) in various forms, including pneumonitis, eosinophilic pneumonia, inflammatory pulmonary nodules, and ILD with granulomatous features [9]. Risk factors for fluoxetine-induced ILD may include long-term therapy, female sex, and older age. Clinicians should maintain a high index of suspicion for drug-induced ILD in patients with new-onset respiratory symptoms, particularly when other etiologies have been ruled out [9]. 

Additionally, fluoxetine has been associated with hypersensitivity pneumonitis, phospholipidosis, and eosinophilic pleural effusion [10,12]. Fluoxetine-induced inflammatory pulmonary nodules with noncaseating giant cell granulomas and non-necrotizing vasculitis have also been reported [13]. Clinicians should therefore recognize the possibility of fluoxetine-induced ILD in patients presenting with new-onset pulmonary symptoms.

Paroxetine

Paroxetine is a selective and potent inhibitor of serotonin reuptake, with a relatively high rate of treatment discontinuation due to its strength and side effect profile. The drug has a mean half-life of approximately 21 hours [14]. 

Paroxetine overdose has been reported to lead to fatal ARDS. It is postulated that this could lead to alveolar damage, hence respiratory failure. A dose as low as 400 mg has been reported to have a fatal outcome [15]. No specific antidote to an overdose of paroxetine currently exists [16]. 

In addition to risks in overdose, paroxetine use during pregnancy has been linked to developmental complications, including persistent pulmonary hypertension of the newborn (PPHN). Based on findings from a 2006 study, the US Food and Drug Administration (FDA) issued a public advisory in 2011 regarding the potential link between SSRI exposure during pregnancy and the development of PPHN [17].

Sertraline 

Sertraline works by increasing serotonergic activity and has an elimination half-life of approximately 26 hours [18]. 

Most overdose cases involve the ingestion of sertraline combined with other drugs, with the most commonly observed side effects generally being non-fatal [19].

In one case, a patient developed a dry cough and hypoxia several weeks after initiating sertraline therapy. Chest computed tomography (CT) imaging revealed diffuse ground-glass opacities and mediastinal lymphadenopathy. After ruling out infectious etiologies, a diagnosis of sertraline-induced ILD was established. The condition resolved following the discontinuation of sertraline and the initiation of corticosteroid therapy [20]. 

The pathogenesis of sertraline-induced ILD is thought to involve direct cytotoxic effects, immune-mediated reactions, and individual variability in drug metabolism and clearance [20]. 

A study analyzing 20 confirmed cases of sertraline-induced ILD, verified through imaging and/or biopsy, found that the most frequently reported symptoms were dyspnea and cough [21].

Due to the unpredictable nature of sertraline-induced ILD, it is prudent to closely monitor pulmonary symptoms and consider imaging if dictated by symptomatology.

Escitalopram

Escitalopram is considered the most potent SSRI [22]. Due to its high potency, it exhibits a relatively rapid onset of action. Escitalopram is primarily metabolized by the liver, and in individuals with hepatic impairment, its elimination half-life is significantly prolonged and nearly doubled.

A case report described a fatal outcome after combined aspiration of food and escitalopram. In this instance, escitalopram dissolved in liquified food was administered to a nursing home resident with dysphagia, which was then found to be lethal. Forensic reports revealed toxic levels of the drug in blood and lung tissues. Autopsy findings included alveolar hemorrhage, diffuse arteriolar microthromboembolism, alveolar edema, and pulmonary vasculopathy. The fatal toxicity was attributed to enhanced systemic absorption through the alveolar-capillary interface, effectively bypassing hepatic first-pass metabolism [23].

SSRIs, including escitalopram, are known to impair platelet function, increasing the risk of bleeding complications [24]. Conversely, there is a case report of pulmonary embolism without any major risk factor in a patient who was started on escitalopram [21].

Fluvoxamine

Fluvoxamine is an SSRI with minimal activity on norepinephrine and dopamine reuptake. It is not approved for use in pediatric populations due to an increased risk of suicidal behavior in children and young adults compared to placebo. Fluvoxamine has an elimination half-life of approximately 16 hours [25]. 

A case of fluvoxamine overdose resulting in aspiration and ARDS was reported in a 30-year-old female. The patient presented to the emergency department with generalized tonic-clonic seizures and vomiting. She subsequently developed acute respiratory failure secondary to severe ARDS, which was attributed to fluvoxamine toxicity. The severity of her condition required extracorporeal membrane oxygenation (ECMO) support as part of her treatment [26].

Atypical agents

Atypical antidepressants exert their effects by modulating various neurotransmitters, including serotonin, dopamine, and norepinephrine. They are considered the second-line therapy for patients who are unable to tolerate traditional SSRIs [27].

Bupropion

Bupropion is a norepinephrine and dopamine reuptake inhibitor (NDRI) that exerts anti-craving and anti-withdrawal effects by increasing dopaminergic and noradrenergic activity [28,29]. Due to these properties, it is widely used as a smoking cessation aid. In combination with naltrexone, bupropion is also approved for weight management, where it is thought to act on hypothalamic pro-opiomelanocortin (POMC) neurons, contributing to reduced food intake. The drug has an elimination half-life of approximately 24 hours.

Bupropion overdose is known to lower the seizure threshold and can precipitate seizures, particularly at high doses.

Cases of diffuse alveolar hemorrhage (DAH) following the inhalation of bupropion have been documented, with noted improvement after the administration of systemic corticosteroids [30,31]. Additionally, a case of acute respiratory failure due to non-cardiogenic pulmonary edema in a pediatric patient following bupropion overdose has also been reported.

Serotonin modulators

Serotonin modulators act as agonists and antagonists at postsynaptic serotonin receptors and inhibit the reuptake of postsynaptic serotonin [32]. These agents are often prescribed as alternative antidepressants for patients who do not respond to or cannot tolerate SSRIs or SNRIs. 

Trazodone

Trazodone is a serotonin reuptake inhibitor that antagonizes histamine (H1) and alpha-1-adrenergic receptors [33]. It has a plasma elimination half-life of approximately seven hours. Notably, there is no specific antidote available for trazodone overdose. 

A case of acute respiratory failure and eosinophilic pneumonia associated with trazodone use has been documented [34]; the diagnosis was confirmed through bronchoscopy and bronchoalveolar lavage. Another reported a patient presenting with pulmonary symptoms, peripheral eosinophilia, and radiographic infiltrates, which resolved upon the discontinuation of trazodone.

Vortioxetine

Vortioxetine is a serotonin modulator and stimulator (SMS) with a half-life of approximately 66 hours. It carries an FDA black box warning for the risk of serotonin syndrome, particularly combined with other serotonergic agents [35]. 

Vortioxetine has been associated with acute eosinophilic pneumonia-like symptoms. One case report described a patient who developed fatigue, loss of appetite, fever, and a productive cough over the course of one week. The condition progressed to acute hypoxemic respiratory failure, requiring high-flow oxygen therapy and intensive care unit (ICU) admission for supportive care [36].

Serotonin-norepinephrine reuptake inhibitors (SNRIs)

SNRIs work by blocking the reuptake of serotonin and norepinephrine, thereby increasing their levels in the synapse. This enhances the transmission of these neurotransmitters [37]. 

Duloxetine

Duloxetine is an SNRI primarily metabolized in the liver, with a half-life of approximately 12 hours. Overdose toxicity has been reported at doses around 1000 mg, commonly presenting with symptoms such as somnolence, serotonin syndrome, and coma. There is no specific antidote, and duloxetine is not effectively cleared by hemodialysis [38].

A case of eosinophilic pneumonia associated with duloxetine use has been reported [39]. The patient presented with elevated eosinophil counts, fever, and pulmonary infiltrates on chest X-ray consistent with eosinophilic pneumonia. Symptoms resolved after the discontinuation of the drug, and eosinophilia subsided. 

Additionally, an overdose-related fatality was documented in an elderly woman; an acute massive pulmonary thromboembolism was identified as the cause of death following duloxetine ingestion [39].

Venlafaxine 

Venlafaxine is an SNRI that has a relatively short elimination half-life of approximately five hours [40]. Compared to other antidepressants, venlafaxine overdose carries a higher risk of fatal outcomes than SSRIs, though it remains less lethal than tricyclic antidepressants (TCAs) [41].

Reports have linked venlafaxine to nonspecific interstitial pneumonia (NSIP), with clinical and radiographic improvement observed following venlafaxine discontinuation and the initiation of corticosteroid therapy [42]. Additionally, cases of acute eosinophilic pneumonia presenting with respiratory insufficiency have been reported. 

These cases demonstrated a rapid resolution upon treatment with corticosteroids, further supporting an association between venlafaxine and pulmonary toxicity [43].

Desvenlafaxine 

Desvenlafaxine, the primary active metabolite of venlafaxine, is predominantly excreted via the urine and has a mean elimination half-life of approximately 11 hours.

A case report described a patient who developed bronchitis-like symptoms and mild persistent dyspnea following an increased dose of desvenlafaxine. Chest CT imaging revealed upper lobe reticular opacities, consistent with NSIP or hypersensitivity pneumonitis. 

Pulmonary function testing showed a persistent restrictive pattern. The patient's symptoms stabilized after the discontinuation of desvenlafaxine and the initiation of steroid therapy combined with mycophenolate mofetil. Follow-up at one month and again at one year showed no further progression of pulmonary symptoms [44].

Milnacipran

Milnacipran is an SNRI approved for the treatment of MDD and fibromyalgia due to its dual mechanism of action. The medication has a half-life of approximately 6-8 hours [45].

A case of milnacipran overdose was associated with the development of acute hypoxic respiratory failure, necessitating mechanical ventilation. The patient also experienced transient heart failure and hypotension, which required vasopressor support. Following the discontinuation of milnacipran, resolution of cardiac dysfunction was confirmed through a follow-up echocardiogram [46].

Tricyclics

TCAs function by inhibiting the reuptake of serotonin and norepinephrine, thereby increasing synaptic concentrations of these neurotransmitters [47]. TCA overdose has been associated with ARDS-like symptoms, likely due to their cardiotoxic and central nervous system depressant effects.

Amitriptyline 

Amitriptyline increases synaptic concentrations of amines such as norepinephrine and serotonin by inhibiting their reuptake in the brain. The drug is rapidly absorbed after oral administration, with a bioavailability of approximately 60% and an elimination half-life of about 25 hours. Ingestion of 750 mg or more in healthy adults can result in severe toxicity, presenting with hypotension, confusion, seizures, mydriasis, and other ocular disturbances [48].

Acute amitriptyline overdose has been associated with pulmonary edema, while chronic use may lead to hypoxia-induced pulmonary hypertension, potentially progressing to right heart failure [47].

Additionally, a case of eosinophilic pneumonia was reported in a dialysis-dependent end-stage renal disease patient receiving amitriptyline [49]. The condition resolved following discontinuation, suggesting a potential causal relationship.

Desipramine

Desipramine is a TCA that primarily inhibits the reuptake of norepinephrine. It has a half-life of approximately seven hours and is primarily eliminated via the kidneys. Common adverse effects include sedation, hypotension, blurred vision, dry mouth, constipation, and urinary retention. Cardiotoxicity may occur with high doses of desipramine [50].

Desipramine overdose has been reported to cause acute hypoxic respiratory failure secondary to severe pulmonary edema, eventually progressing to ARDS. The lung injury is thought to result from a combination of direct toxic effects on pulmonary parenchyma, aspiration, and metabolic acidosis. Clinical improvement was observed following intensive care management [51].

Nortriptyline

Nortriptyline, the active metabolite of amitriptyline, exerts its effects by blocking the reuptake of serotonin and norepinephrine at neuronal synapses [52]. It has an average elimination half-life of around 26 hours. Symptoms of overdose may include cardiac arrhythmias, profound hypotension, congestive heart failure, pulmonary edema, seizures, and central nervous system depression.

In one reported case, aspiration of nortriptyline led to worsening respiratory distress, ultimately necessitating mechanical ventilation in an ICU. Bronchoscopic examination revealed airway inflammation and edema, suggesting that lung injury may have resulted from aspiration-related damage.

In another case, pulmonary edema was identified on imaging, an uncommon and rarely documented adverse effect. It was postulated that this reaction might be due to heightened sensitivity to external sympathomimetic agents [53].

Imipramine

Imipramine is extensively metabolized by the liver, with a half-life of approximately 12 hours. Toxicity has been observed in humans at doses exceeding 5 mg/kg [54].

A case report described a patient who developed ILD after taking imipramine consistently for 25 years. The patient presented with progressive dyspnea, and diagnostic evaluations, including pulmonary function tests (PFTs) and high-resolution computed tomography (HRCT), revealed features consistent with restrictive lung disease, supporting the diagnosis of imipramine-induced ILD [55]. 

Monoamine oxidase inhibitors (MAOIs)

MAOIs were among the first antidepressants introduced, but are no longer considered first-line treatments for depression due to their side effect profile. They are typically reserved for treatment-resistant cases when other antidepressant classes have failed.

A study reported that patients receiving MAOIs exhibited a higher incidence of asthmatic symptoms compared to those in the placebo group, suggesting a potential respiratory side effect associated with this drug class [56]. 

Phenelzine 

Phenelzine increases brain levels of gamma-aminobutyric acid (GABA) and alanine (ALA) by inhibiting the enzymes responsible for their metabolism. It is rapidly absorbed in the gastrointestinal tract and has a half-life of approximately 11 hours. Phenelzine toxicity may present as asymptomatic elevations in aminotransferase levels or, less commonly, as clinically apparent liver injury, typically occurring within 1-3 months of high-dose therapy [57].

A lupus-like reaction was reported in a patient treated with phenelzine sulfate for depression. Imaging also revealed pulmonary edema, an adverse event not previously associated with this medication. This reaction was hypothesized to result from hypersensitivity to exogenous sympathomimetic agents [58].

## Conclusions

As global antidepressant use continues to rise, there is an increasing need to monitor for pulmonary toxicity, an underrecognized but significant risk associated with these medications.

Adverse pulmonary effects occur at both therapeutic and overdose levels, underscoring the need for early detection and timely intervention. Clinicians should maintain a high index of suspicion for new-onset respiratory symptoms, such as cough or dyspnea, following the initiation of antidepressant therapy. Vigilance should extend beyond the initial treatment period, as delayed or cumulative pulmonary toxicity may occur with long-term use. In overdose cases, healthcare providers must proactively assess for respiratory complications. Ultimately, recognizing and managing antidepressant-induced lung toxicity is essential to improving patient outcomes and preventing long-term respiratory morbidity.
